# Influence of the Ag concentration on the medium-range order in a CuZrAlAg bulk metallic glass

**DOI:** 10.1038/srep44903

**Published:** 2017-03-21

**Authors:** C. Gammer, B. Escher, C. Ebner, A. M. Minor, H. P. Karnthaler, J. Eckert, S. Pauly, C. Rentenberger

**Affiliations:** 1 Erich Schmid Institute of Materials Science, Austrian Academy of Sciences, Jahnstraße 12, 8700 Leoben, Austria; 2 IFW Dresden, Institute for Complex Materials, Helmholtzstraße 20, 01069 Dresden, Germany; 3 University of Vienna, Faculty of Physics, Boltzmanngasse 5, 1090 Wien, Austria; 4 National Center for Electron Microscopy, Molecular Foundry, Lawrence Berkeley National Laboratory, Berkeley, CA, USA; 5 Department of Materials Science and Engineering, University of California, Berkeley, CA, USA; 6 Department Materials Physics, Montanuniversität Leoben, Jahnstraße 12, 8700 Leoben, Austria

## Abstract

Fluctuation electron microscopy of bulk metallic glasses of CuZrAl(Ag) demonstrates that medium-range order is sensitive to minor compositional changes. By analyzing nanodiffraction patterns medium-range order is detected with crystal-like motifs based on the B2 CuZr structure and its distorted structures resembling the martensitic ones. This result demonstrates some structural homology between the metallic glass and its high temperature crystalline phase. The amount of medium-range order seems slightly affected with increasing Ag concentration (0, 2, 5 at.%) but the structural motifs of the medium-range ordered clusters become more diverse at the highest Ag concentration. The decrease of dominant clusters is consistent with the destabilization of the B2 structure measured by calorimetry and accounts for the increased glass-forming ability.

Bulk metallic glasses (BMGs) exhibit distinct mechanical properties such as high strength combined with a large elastic strain but limited ductility owing to the lack of a long-range structural order^1^. Despite recent advances, the understanding of the local structure of BMG and its influence on the macroscopic physical properties still remains a fundamental challenge. Icosahedral clusters identified in molecular dynamic (MD) simulations are frequently claimed to constitute the short-range order^2^. In addition, correlations on a longer length-scale (0.5–3 nm) termed medium-range order (MRO) are expected to be present in BMGs^3^. Most of the information on MRO in amorphous materials relies on simulations because standard scattering experiments average over volumes considerably larger than the size of the structural fluctuations.

A promising experimental approach to measure structural order at this length scale is fluctuation electron microscopy (FEM)^4^,^5^,^6^. FEM can be carried out in the scanning transmission electron microscope (STEM) by computing the intensity variance of spatially resolved nanodiffraction patterns as a function of the scattering vector *k* for different resolutions *R*^4^. The peaks in the resulting normalized intensity variance *V(k)* are a fingerprint of the structural correlations in the sample. Varying the resolution allows to get an insight into the length scale of the correlations. FEM has been shown to be sensitive to the MRO in BMGs such as interconnected clusters or crystal-like regions^7^.

CuZr based metallic glasses are promising candidates for structural applications due to their relatively low cost and desirable mechanical properties^8^. It was shown that minor additions of alloying elements can have a dramatic effect on the phase stability of BMG and its mechanical and physical properties^9^,^10^. Depending on the element, alloying of CuZr based BMG can promote the precipitation of austenitic B2 CuZr crystals forming composites with enhanced ductility^11^,^12^,^13^ or can lead to a significant enhancement of the glass-forming ability (GFA)^14^,^15^. A structural understanding of the reason for the pronounced effect of minor alloying elements on the formation and properties of BMG is still incomplete. Therefore, in the present work we present a systematic FEM study of CuZrAl BMG with varying Ag addition and try to link the evolution of local ordering as a function of the composition with GFA, phase stability and activation energy for crystallization.

## Results

**Figure 1:**
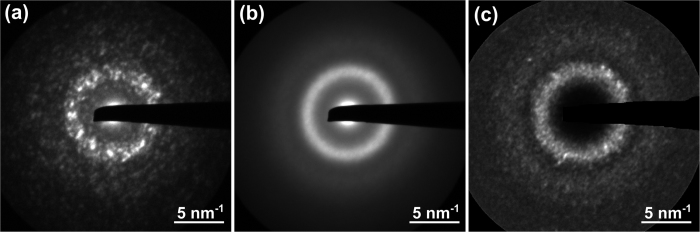
Example of the nanodiffraction FEM. (**a**) Representative nanodiffraction pattern extracted from the dataset acquired on the Ag-free Cu_47.5_Zr_47.5_Al_5_ alloy with a beam size of 1.2 nm. (**b**) Average of all nanodiffraction patterns in the dataset. (**c**) Normalized intensity variance calculated for each pixel across the entire dataset.

**Figure 2:**
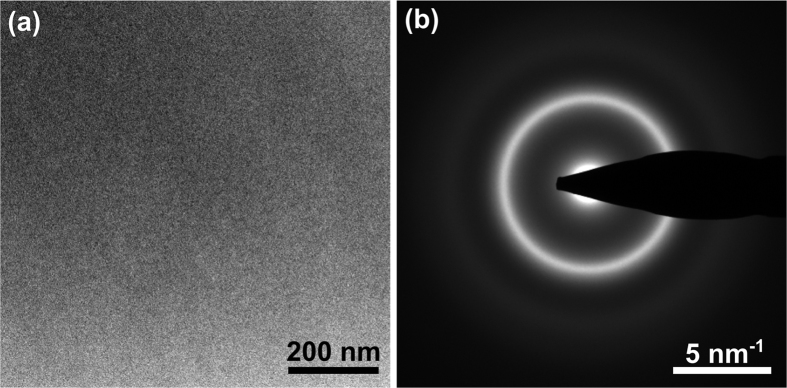
TEM images of Cu_46.5_Zr_46.5_Al_5_Ag_2_ BMG. Bright field-image (**a**) and the corresponding selected area diffraction pattern (**b**) taken from a large area of the Cu_46.5_Zr_46.5_Al_5_Ag_2_ BMG sample reveal a homogeneous amorphous structure without the presence of nanocrystals.

**Figure 3:**
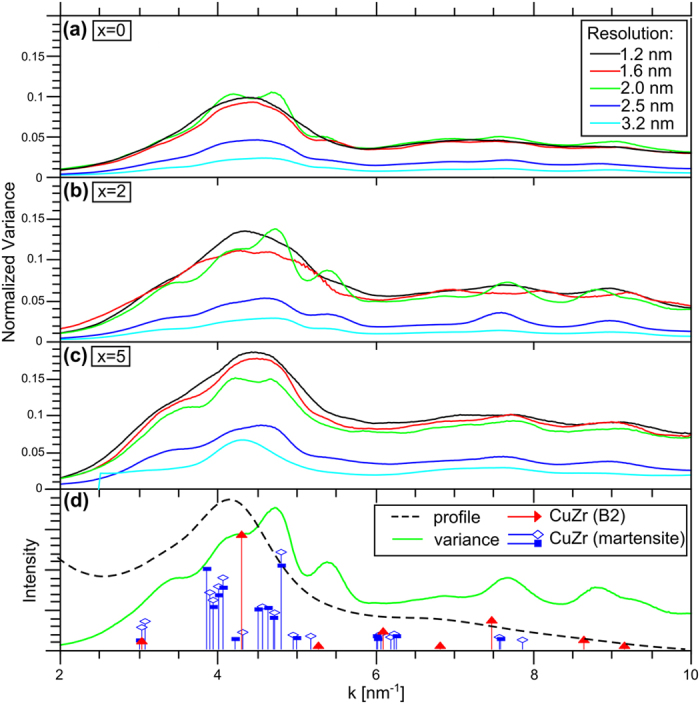
Normalized variance for the various CuZrAl(Ag) BMGs. (**a**–**c**) Normalized variance profiles *V(k*) determined by azimuthal averaging of the 2D normalized variance patterns are shown for different resolutions as indicated by their colors. Peaks are visible in the variance plots, indicating the presence of medium-range order. Results for the different BMGs are presented. (**d**) The profile obtained from the average diffraction pattern is shown on top of a normalized variance curve taken from (**b**). For comparison, the peak positions for B2 CuZr and its martensites are indicated.

Figure 1a shows a typical nanodiffraction pattern using a probe size of 1.2 nm for the Cu_47.5_Zr_47.5_Al_5_ BMG. Averaging over all 225 nanodiffraction patterns in the dataset results in an image that resembles a conventional diffraction pattern (cf. Fig. 1b). Figure 1c shows the corresponding 2D normalized variance pattern calculated by computing the normalized variance of the intensity across all nanodiffraction patterns in the dataset for each pixel in the diffraction pattern. All data evaluation was carried out using custom DigitalMicrograph™ scripts. In order to demonstrate that the BMG samples are homogenously amorphous bright-field images and selected area electron diffraction patterns were acquired in addition to the series of nanodiffraction patterns. As an example Fig. 2 shows characteristic images of the BMG sample containing 2 at.% Ag. Despite the fact that the variance curve of this sample shows the most pronounced peaks (cf. Fig. 3), no indication of nanocrystals was found in the investigated area.

Figure 3 shows the FEM profiles of the three different (Cu_0.5_Zr_0.5_)_95−x_Al_5_Ag_x_ (x = 0, 2, 5) alloys using different probe sizes. The normalized variance profile *V(k*) was obtained by azimuthal average of the 2D variance pattern as the one given in Fig. 1c. Center determination and azimuthal averaging was carried out using the PASAD tools^16^. The variance curve *V(k*) of the Cu_47.5_Zr_47.5_Al_5_ sample at the highest resolution *R* = 1.2 nm shows a pronounced first broad peak at about 4.5 nm^−1^ (cf. Fig. 3a). The position is different to that of the maximum of the mean intensity *I(k*) at *k* = 4.2 nm^−1^ (cf. Fig. 3d dashed curve). By increasing the probe size, the broad peak in *V(k*) splits into two sub-peaks with a similar variance level. The emergence of sub-peaks that are especially pronounced at a resolution of 2 nm indicates structural fluctuations (at that particular length scale) that scatter into the corresponding *k* directions. Caused by peak splitting, the position of the first sub-peak in *V(k*) at *k* = 4.15 nm^−1^ coincides with the position of the maximum mean intensity at *k* = 4.2 nm^−1^ whereas the second peak is found at *k* = 4.7 nm^−1^. The variance hump at *k* = 3.5 nm^−1^ correlates with the oxide layer at the surface^17^. Additional peaks at higher *k* values can be detected at *k* = 5.35, 6.9, 7.65 and 8.8 nm^−1^. At larger *R* values corresponding to larger scattering volumes, the sub-peaks merge again and the overall magnitude of *V(k*) as well as its *k*-dependence decrease. Figure 3b,c show the FEM profiles of the samples with 2 and 5 at.% Ag, respectively. The corresponding *V(k*) curves obtained from patterns taken at different resolutions show the same trends as described for the Ag-free sample. Nevertheless, with increasing Ag content the variance values increase. The numerous peaks are especially pronounced for the sample containing 2 at.% Ag.

**Figure 4:**
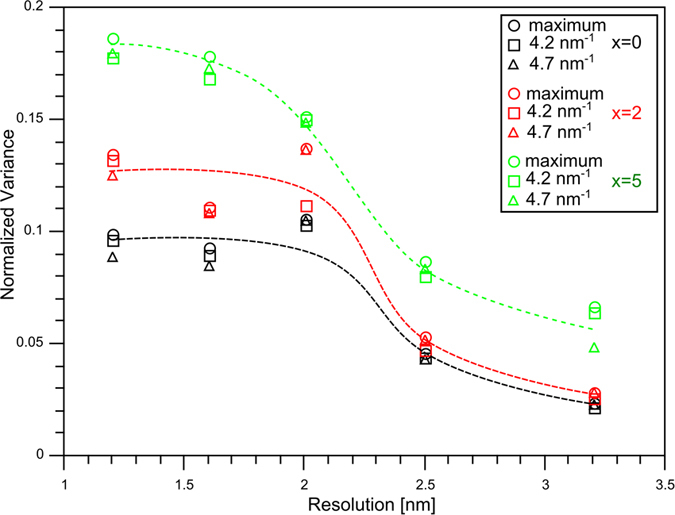
Comparison of the normalized variance as a function of composition and resolution. Values at three different reciprocal vectors *k* were extracted from the variance plots and shown as a function of resolution: the maximum and the values at 4.2 and 4.7 nm^−1^. A comparison for the various compositions shows that the normalized variance increases with increasing Ag concentration. The pronounced drop at about 2 nm for all samples indicates similar MRO length scale. The dashed lines serve as a guide to the eye.

Figure 4 shows the values for the normalized variance peaks as a function of resolution *R*. Three values are extracted from the variance plots *V(k*): the values at max(*V(k*)) corresponding to *k* ~ 4.5 nm^−1^ as well as those at the sub-peak positions *k* = 4.2 and 4.7 nm^−1^. The variance is rather similar for resolutions in the range of 1.2 to 2 nm and significantly drops at larger resolutions. The pronounced drop between 2 and 2.5 nm indicates that the scale of the structural fluctuations is in the range of 2 nm for all compositions. A comparison of the samples with 0, 2 and 5 at.% Ag reveals that the normalized variance increases with increasing Ag concentration.

**Figure 5:**
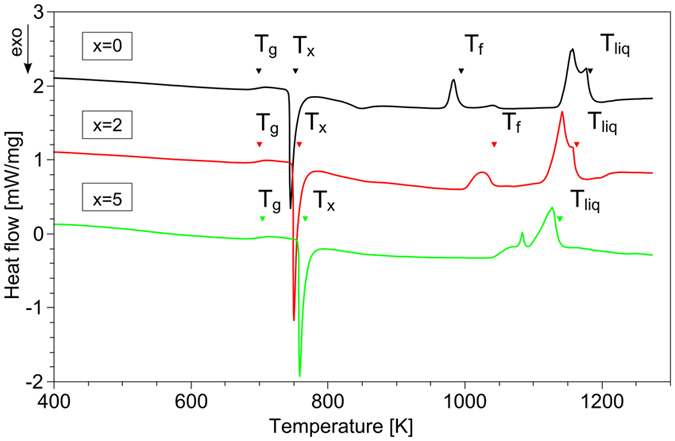
Differential calorimetry curves (heating rate 20 K/min) of the various CuZrAl(Ag) BMG (for clarity the curves are shifted vertically). The glass transition temperature T_g_, the crystallization temperature T_x_, the final B2 transformation temperature T_f_ and the liquidus temperature T_liq_ are indicated. The decreasing temperature interval T_liq_-T_f_ with increasing Ag content indicates destabilization of the B2 phase.

The thermal stability behavior of the three alloys is shown in Fig. 5 by the corresponding high-temperature DSC scans. The glass transition and the crystallization events can be clearly seen by their endothermic and exothermic heat flow events, respectively. The supercooled liquid region increases slightly after the addition of Ag since *T*_x_ increases and *T*_*g*_ is almost unaffected. Endothermic events at higher temperatures are correlated with the occurrence of the B2 phase transformation that is finished at the final temperature *T*_f_^18^. The increase of *T*_f_ indicates destabilization of the B2 phase with Ag addition and is reflected in the corresponding K-parameter that was defined to predict the formation of BMG composites with good deformability^18^. All data evaluated from DSC scans are given in Table 1.

## Discussion

In general, a high normalized variance *V(k*) as shown in Fig. 3 indicates a large degree of structural fluctuations^19^. We also find multiple peaks in the variance curve of all CuZrAl-based samples at the optimized resolution although they do not exist in *I(k*). A high normalized variance in combination with the occurrence of pronounced peaks at certain *k*-values strongly indicates that crystal-like clusters on the MRO scale are present in the glass and they are expected to be homogeneously distributed over the sample volume. The MRO crystal-like clusters are considered to have a length scale of about 2 nm since both pronounced sub-peaks of *V(k*) are observed at a resolution of 2 nm and the normalized variance decays considerably above 2 nm (cf. Figs 3 and 4). Using the paracrystallite model with a characteristic length at which the correlation between atom-pairs decays^20^ a similar MRO size of 1.6 nm can be deduced from the variable resolution FEM data of the Cu_45_Zr_45_Al_5_Ag_5_ sample. For the other two alloys this model fails due to the presence of the pronounced sub-peaks showing high variance values.

In order to obtain structural information from the FEM results, the position of the maxima in *V(k*) are analyzed first. The broad peak in *V(k*) calculated from the FEM results of the Cu_47.5_Zr_47.5_Al_5_ sample is split into sub-peaks located at about 4.2 and 4.7 nm^−1^ by adapting the probe size (lateral resolution). The positions of these sub-peaks are similar to those measured by FEM in Cu_65.5_Zr_34.5_ by Hwang *et al*.^17^: *k* = 4.3, 4.7 nm^−1^. Further FEM studies on Zr_50_Cu_45_Al_5_ and Zr_50_Cu_35_Al_15_ BMGs revealed one variance peak near *k* = 4.1 nm^−1^ with shoulders at lower or higher *k* values^19^,^21^. By combining the FEM results with reverse Monte Carlo modelling icosahedral-like as well as crystal-like clusters represented by different k-values were identified in ref. 21. The icosahedral-like clusters were associated with the shoulder present at *k* = 3.7 nm^−1 ^ in ref. 21. Therefore, the pronounced peaks in *V(k*) observed in the present study are interpreted as a consequence of the presence of crystal-like clusters. Their enhanced visibility compared to refs 19 and 21 might be a consequence of various structural motifs combined with enhanced internal order.

A comparison of the peak positions in *V(k*) of amorphous Cu_47.5_Zr_47.5_Al_5_ with reciprocal vectors of different binary (Cu-Zr, Cu-Al and Zr-Al) and ternary crystalline phases indicates the dominance of crystal-like cluster types based on the B2 CuZr structure and its martensites. The occurrence of the first two sub-peaks present in *V(k*) at k ~ 4.2 and 4.7 nm^−1^ can be described by clusters based on the two monoclinic martensitic phases whereas the positions of the peaks at high *k* values agree well with B2 like CuZr clusters (cf. Fig. 3d). The agreement is within the experimental error of the absolute value of the camera length of a TEM (up to 3%). The crystal structures of B2 CuZr and of the B19’ and B33 martensites were taken from refs 22 and 23. At first sight the presence of martensitic clusters is not expected to occur since the transformation from the B2 structure to the monoclinic martensitic structure during cooling to room temperature is usually suppressed in small sized clusters due to the decreased transformation temperature^15^,^24^. However, high internal stresses in the BMG could induce the martensitic transformation similar to loaded nanograined NiTi^25^ or stabilize distorted structures resembling the martensitic phase. Therefore, it is concluded that during casting of Cu_47.5_Zr_47.5_Al_5_ from the melt crystal-like clusters with structural motifs based on the B2 phase and its martensites are formed in the amorphous material and are homogeneously distributed. This structural homology of the BMG with the high-temperature crystalline phase is consistent with the basic concept of principal clusters in BMGs derived from crystalline phases^26^. Based on B2 CuZr the principal clusters are [Cu-Zr_8_Cu_6_] and [Zr-Cu_8_Zr_6_] with the central atom separated from its nearest neighbours by a hyphen. It is important to point out that the structural motifs found by FEM differ from the low-temperature equilibrium phases Cu_10_Zr_7_ and CuZr_2_ formed by devitrification of the glassy state^18^. Based on the structures and the negative heat of mixing, the crystal-like MRO clusters seem to be dominated by Cu-Zr bonds. It is worth mentioning that the first peak at 4.2 nm^−1^ is consistent with some CuZr-based structures^22^,^27^ indicating the importance of the Cu-Zr bonds. It should also be noted that the amorphous zirconium oxide layer formed on CuZr metallic glasses has a strong contribution to *V(k*). In the present work we minimized oxidation by avoiding contact with water humidity and selecting appropriate areas of the sample in the TEM, leaving only a hump at k = 3.5 nm^−1^ simplifying the interpretation of the *V(k*) curve. The shape of the hump is different for the various samples since the oxidation layer was differently pronounced.

The presence of crystal-like clusters with high internal order seems to be characteristic for the amorphous structure of our Cu_47.5_Zr_47.5_Al_5_ glass. They might form because the actual cooling rate is rather close to the critical cooling rate for vitrification, that is expected to promote nucleation and subsequent freezing-in of B2 and martensite based clusters during cooling. At high experimental cooling rates, as for melt spinning (10^4^–10^6^ K/s^28^), the fraction of icosahedral-like clusters has been found to be increased at the expense of crystal-like MRO clusters as indicated by the less pronounced peaks in *V(k*)^19^,^21^. At the extremely high cooling rates (10^10^–10^12^ K/s) as in the case of molecular dynamics (MD) simulations the structure is dominated by Cu-centred and Al-centred clusters with a special role of clusters with icosahedral order^29^. Nevertheless, MD simulations analyzed by Wu *et al*. show also the presence of hidden order in metallic glasses inherited from fcc and bcc order^30^.

With the addition of Ag the content of Cu and Zr was reduced according to (Cu_0.5_Zr_0.5_)_95−x_Al_5_Ag_x_ with x = 2 and 5. The change of the alloy composition leads to a general increase of the normalized variance V(k) (cf. Figs 3 and 4). Due to the positive heat of mixing of Cu and Ag, they tend to avoid the formation of bonds. Therefore, it is expected that at low Ag concentrations Cu atoms in crystal-like MRO clusters are substituted by Ag atoms to form Ag-Zr, Ag-Al or Ag-Ag bonds of the same MRO cluster motifs. As a result the signature of the variance curve (as a function of k and R) is unaffected but the variance increases due to the presence of Ag as a strong scatterer. The replacement of Cu by Ag and the formation of Ag-Ag pairs are consistent with ab-initio simulations^29^.

At 5 at.% Ag concentration the visibility of the different sub-peaks corresponding to special crystal-like clusters decreases although the absolute variance level increases. This behavior indicates that the characteristic of the MRO structure becomes more hidden in the variance curve with the addition of 5 at.% Ag although the tendency of forming MRO clusters is still high. Therefore, it can be assumed that the dominance of the B2 and martensite like MRO motifs of the clusters decreases and other motifs partially take their place. The reduced dominance of the B2 like MRO clusters in the BMG containing 5 at.% Ag is again consistent with the principal cluster model^26^ since the structural change in the BMG correlates with the reduced B2 phase stability revealed from experimental DSC results: the high-temperature crystalline B2 phase is less prone to form when the Ag concentration increases (cf. Fig. 5). The high variance level measured in this sample is mainly attributed to the presence of Ag since the amount of MRO seems only slightly affected as indicated by the MRO length scale insensitive to the Ag concentration (cf. Fig. 4). The formation of MRO clusters can also be accompanied by chemical heterogeneities. The development of chemical heterogeneities in the form of Ag-dominated clusters is supported experimentally by the use of Atom Probe Tomography of Cu-Zr-Al-Ag BMG samples prepared by copper mold casting^31^. The lack of phase separation in rapidly quenched ribbons of Cu_40_Zr_40_Al_10_Ag_10_^32^ may point to a cooling-rate dependency.

The glass-forming ability (GFA) of a BMG is a consequence of the competition between vitrification and crystallization upon cooling. By analyzing the structure of the amorphous state we conclude that during cooling MRO clusters with motifs based on B2 and martensite structures are formed in Cu_47.5_Zr_47.5_Al_5_ BMG. With the addition of Ag an increase of the GFA for CuZrAlAg alloys was shown^33^,^34^,^35^. This is confirmed by the increase of the thermodynamic parameter γ = T_x_/(T_g_ + T_liq_) (cf. Table 1) that describes the GFA^36^. In addition to simple thermodynamic parameters, it was suggested that the GFA is strongly determined by the local structure such as efficient packing of icosahedral clusters or MRO^3^,^14^,^21^,^30^. The present experimental results based on FEM are interpreted as a weak increase in MRO and a reduced dominance of a special structural motif forming the MRO. The latter is also supported by MD simulations showing higher variability of hidden order inherited from crystalline order as the number of chemical components of glassy alloys increases^30^. Therefore, it is concluded that the increased GFA by adding Ag is a structural consequence of the beginning destabilization of the B2 structure and the resultant competition of the formation of various structural motifs showing MRO (e.g. motifs based on the low temperature equilibrium structures Cu_10_Zr_7_ and CuZr_2_ as it would be suggested by the principal cluster model). In a nutshell, the higher complexity of crystallization upon cooling in Cu_45_Zr_45_Al_5_Ag_5_ causes an increased GFA. This basic concept has been suggested by several authors, e.g. refs 30 and 37.

The discussed change of the dominant MRO motifs by adding Ag is consistent with the decrease of the activation energy for crystallization upon heating (cf. Table 1). Since Cu_47.5_Zr_47.5_Al_5_ BMG crystallizes into Cu_10_Zr_7_ and CuZr_2_^18^, the MRO structure of the Cu_47.5_Zr_47.5_Al_5_ metallic glass with dominant B2-like motifs is far away from the equilibrium crystal structures and the activation energy for crystallization is high. The destabilization of the B2 structure with increasing Ag content suggests that other crystal-like motifs become more dominant (e.g. similar to the equilibrium crystal structure Cu_10_Zr_7_ and CuZr_2_) leading to the reduction of the activation energy for crystallization upon heating.

### Summary

The present work presents systematic FEM studies of CuZrAl(Ag) bulk metallic glasses with a wide range of spatial resolutions by the use of different STEM probe sizes. By analyzing the normalized variance curve *V(k*) calculated from the intensities of nanodiffraction patterns we find a pronounced MRO with crystal-like motifs based on the high-temperature crystalline B2 CuZr structure and its distorted structure resembling the martensitic ones. The presence of MRO clusters is consistent with the low cooling rate of suction casting compared to melt spinning. By varying the composition an increase of the normalized variance *V(k)* is measured but the MRO length scale seems not affected. Therefore, we propose that only a slight increase of the MRO with increasing Ag addition occurs and the increase of the normalized variance is mainly attributed to the presence of Ag as strong scatterer. At the highest Ag content of 5 at.% the structural motifs forming the MRO clusters becomes more hidden indicating a decrease of the dominant clusters of the B2 and martensitic structures. This result demonstrates that MRO can be sensitive to minor compositional changes. In combination with DSC scans, the increase in GFA with increasing Ag contents is attributed to the lower tendency of forming the B2 structure and the resultant competition of the formation of various MRO clusters based on different motifs.

## Methods

Starting from ingots of the master alloys prepared by arc melting of high-purity elements (Al, Cu: 99.99 wt%, Ag: 99.9 wt% Zr: 99.8 wt%) under a Ti-gettered Ar atmosphere, (Cu_0.5_Zr_0.5_)_95−x_Al_5_Ag_x_ (x = 0, 2, 5) rods with a diameter of 2 mm were cast into Cu-molds by suction-casting (modified Bühler MAM1). Disc shaped TEM samples were cut from the bulk specimens and ground to a thickness of 0.15 mm. The discs were thinned to electron transparency using twin-jet electropolishing (Struers Tenupol-3) and a solution of methanol with 33% nitric acid at −25 °C. This method yielded samples with large electron transparent regions and minimal damage and minimal thickness variation.

All samples were plasma-cleaned prior to the TEM measurements to remove carbon contamination. The TEM investigations were carried out in a FEI Titan microscope operating at 300 kV. Its three-condenser system allows for control of the semi-convergence angle to form rather parallel nanometer-sized electron probes. The TEM was equipped with a field emission gun forming coherent electron probes. The FEM measurements were carried out in STEM microprobe mode using a small condenser aperture (C2 = 40 μm). To study the length-scale of the medium-range order probes with five different sizes ranging from 1.2 to 3.2 nm were used. The semi-convergence angle was varied between 0.96, 0.62 and 0.49 mrad at a fixed spot size of 9 to form probes with a size of 1.2, 1.6 and 2 nm, respectively. To reach larger probe sizes with 2.5 and 3.2 nm, the spot size was changed to values of 7 and 6, respectively while keeping the semi-convergence angle at 0.63 mrad. The probe size was measured in vacuum before and after each experiment using a CCD camera.

Nanodiffraction patterns were acquired from a 15 × 15 raster, yielding 225 diffraction patterns. The step size of 10 nm was chosen to be larger than the probe diameter to minimize any potential beam-induced sample contamination. Nanodiffraction patterns (1024 × 1024 pixel in size) were acquired with a Gatan Orius CCD at binning 2, using an exposure time of 0.5 sec. All areas used for the measurements were carefully checked to make sure that they contained no contamination or crystallites. To ensure that all measurements were taken from regions with equal thickness, the ratio of the intensity for a bright-field image in the region of interest and in vacuum was compared for each of the investigated regions.

Thermal analysis of the different alloys was performed in a high temperature differential scanning calorimeter (DSC) (Netzsch DSC 404 C) and in a conventional DSC (Perkin-Elmer Diamond). Using the Perkin-Elmer Diamond DSC at heating rates ranging from 5 to 300 K/min the glass transition temperatures *T*_*g*_, the crystallization temperatures *T*_*X*_ (onset) and the activation energies for crystallization were obtained. The start (*T*_*s*_) and end (*T*_*f*_) temperatures of the eutectoid transformation (Cu_10_Zr_7_ + CuZr_2_ ↔ B2 CuZr)^18^, as well as the solidus temperature *T*_*sol*_ and the liquidus temperature *T*_*liq*_ were determined from high-temperature measurements in the Netzsch DSC using a heating rate of 20 K/min.

## Additional Information

**How to cite this article:** Gammer, C. *et al*. Influence of the Ag concentration on the medium range order in a CuZrAlAg bulk metallic glass. *Sci. Rep.* **7**, 44903; doi: 10.1038/srep44903 (2017).

**Publisher's note:** Springer Nature remains neutral with regard to jurisdictional claims in published maps and institutional affiliations.

## Figures and Tables

**Table 1:** Characteristic temperatures T_g_, T_x_, T_f_, and T_liq_ the thermodynamic parameters γ, K and the activation energy of crystallization for the different alloys.

	T_g_ [K] ± 1 K	T_x_ [K] ± 1 K	T_f_ [K] ± 1 K	T_liq_[K] ± 1 K	γ = T_x_/(T_g_ + T_liq_) ± 1 × 10^−4^	K = T_f_/T_liq_ ± 2 × 10^−4^	Activation energy [kJ/mol]
Cu_47.5_Zr_47.5_Al_5_	699	753	994	1183	0.399	0.836	407 ± 10
Cu_46.5_Zr_46.5_Al_5_Ag_2_	700	758	1043	1163	0.407	0.897	358 ± 13
Cu_45_Zr_45_Al_5_Ag_5_	704	767	>T_sol_	1139	0.416	~1	325 ± 13
